# Design and Synthesis of Quick Setting Nonswelling Hydrogels via Brush Polymers

**DOI:** 10.1002/advs.202100968

**Published:** 2021-06-20

**Authors:** Fei Jia, Joshua M. Kubiak, Michika Onoda, Yuping Wang, Robert J. Macfarlane

**Affiliations:** ^1^ Department of Materials Science and Engineering Massachusetts Institute of Technology (MIT) 77 Massachusetts Avenue Cambridge MA 02139 USA

**Keywords:** brush polymers, fast‐forming hydrogels, multifunctional crosslinkers, polymer hydrogels, polymer NPs

## Abstract

Brush polymers have emerged as components of novel materials that show huge potential in multiple disciplines and applications, including self‐assembling photonic crystals, drug delivery vectors, biomimetic lubricants, and ultrasoft elastomers. However, an understanding of how this unique topology can affect the properties of highly solvated materials like hydrogels remain under investigated. Here, it is investigated how the high functionality and large overall size of brush polymers enhances the gelation kinetics of low polymer weight percent gels, enabling 100‐fold faster gelation rates and 15‐fold higher stiffness values than gels crosslinked by traditional star polymers of the same composition and polymer chain length. This work demonstrates that brush polymer topology provides a useful means to control gelation kinetics without the need to manipulate polymer composition or crosslinking chemistry. The unique architecture of brush polymers also results in restrained or even nonswelling behavior at different temperatures, regardless of the polymer concentration. Brush polymers therefore are an interesting tool for examining how high‐functionality polymer building blocks can affect structure–property relationships and chemical kinetics in hydrogel materials, and also provide a useful rapidly‐setting hydrogel platform with tunable properties and great potential for multiple material applications.

## Graphical abstract

Hydrogel properties of star polymers and a series of brush polymers with different DPs were compared. The high branch functionality, large overall size, and extended side chains of the brush polymers enable gelation rate of brush polymer hydrogels to be over 100 times faster than 4‐arm star polymer hydrogels with identical chemical composition and show nonswelling property at body temperature.

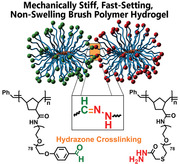

## 1 Introduction

Hydrogels are ubiquitous in both biomedicine (e.g., wound dressings, tissue engineering scaffolds, and surgical adhesives) and device engineering (e.g., electronics coatings, adhesives, and soft robotics).^[^ ^1^, ^2^ ^]^ A broad range of polymer species and network structures have been used to tailor the physical properties and behaviors of these materials to match their desired functions.^[^ ^3^, ^4^ ^]^ In the development of new hydrogels, the ability to use multiple design handles to tune structure–property relationships and a complete understanding of how those handles affect gel behavior are critical. In particular, quick‐setting and nonswelling behaviors are key properties for hydrogels, especially in applications related to biomedicine. Rapid gelation allows gels to be formed directly at the site of use, and nonswelling behaviors can improve biocompatibility when the gels interface with soft or easily damaged tissue types.^[^ ^5^, ^6^, ^7^, ^8^ ^]^ The use of large, high‐functionality polymers (e.g., chitosan, cellulose, and collagen) is a common strategy to prepare such quick‐setting gels, as gelation kinetics are greatly enhanced by the large number of crosslinking sites along the polymer backbone.^[^ ^9^, ^10^ ^]^ However, chain entanglements and strong physical interactions between these large species can make them difficult to dissolve during gel preparation, and gel precursor solutions with large *M*_w_ polymers are often prohibitively viscous.^[^ ^10^, ^11^, ^12^, ^13^, ^14^ ^]^ For nonswelling properties, hydrogels often incorporate thermoresponsive blocks with a lower critical solution temperature (LCST), where shrinking of these LCST blocks compensates for swelling at elevated temperatures.^[^ ^15^ ^]^ An alternative strategy is to introduce additional chemical or physical connections between polymers (e.g., covalent or physical bonds, hydrophobic moieties that aggregate in aqueous solution) to form a more crosslinked network and inhibit swelling.^[^ ^7^, ^16^ ^]^ The major challenge for these strategies is that they require additional screening to ensure that these chemical modifications induce the desired physical characteristics without introducing negative chemical properties or biological interactions. Therefore, fundamental investigations into alternative strategies to induce rapid gelation or to prevent aggressive swelling that do not require altering the chemical composition of the polymer or crosslinking agents would greatly benefit hydrogel research.

**Scheme 1:**
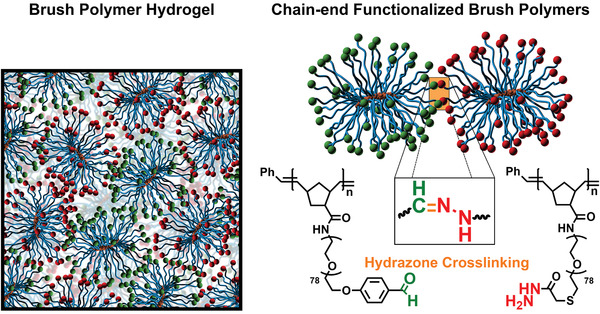
Schematic illustration of brush polymer hydrogel crosslinked via hydrazone formation.

In this work, we examine the use of crosslinkable brush polymers with high functionality (*f*) as a potential design tool for inducing these physical properties in hydrogels (**Scheme** **1**). These experiments aim to provide a more thorough understanding of how these unique polymer topologies affect aspects of gel design like gelation kinetics, mechanical response, and long‐term stability. We show that brush polymers can be used to make quick setting, nonswelling hydrogels with beneficial chemical and physical characteristics and explain how brush architecture induces these unique material properties. The information developed here therefore allows the brush polymer topology to be a useful and interesting tool in the synthesis of advanced hydrogel materials.

A brush polymer is composed of a primary polymer backbone densely grafted with a tunable number of secondary polymer side chains; large branch functionalities can, in principle, be achieved by installing crosslinking groups at the terminus of each secondary chain.^[^ ^17^ ^]^ This topology provides an interesting means of controlling structure–property relationships in gels due to the unique behaviors exhibited by brush polymers compared with their linear or star polymer counterparts. For example, steric hindrance between adjacent side chains forces the polymer brushes into more elongated conformations than typical, gaussian linear polymers. Therefore, the interchain entanglement often observed in linear polymers is greatly reduced, and the viscosity of concentrated polymer solutions is comparatively lower in brush polymer systems than solutions of linear polymers of the same *M*_w_.^[^ ^18^ ^]^ Furthermore, steric crowding should force the brushes into extended conformations both before and during gel formation, thereby minimizing the extent to which the brushes can swell when the gels are subsequently immersed in solution.^[^ ^19^, ^20^ ^]^

## 2 Results and Discussion

**Figure 1:**
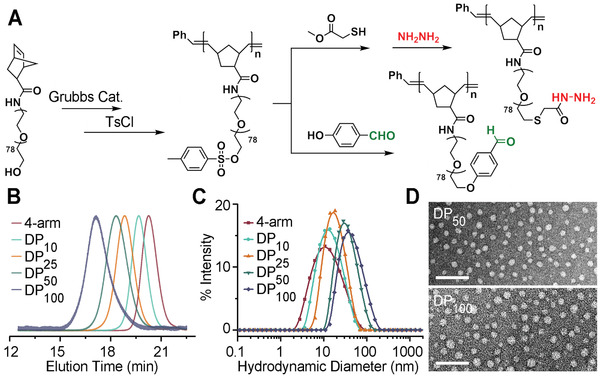
A) Schematic for polymerization and postsynthetic modification of brush polymers. B) GPC chromatograms, and C) intensity‐average hydrodynamic diameter distributions for synthetic polymers with different DPs. D) TEM images of aldehyde modified DP_50_ and DP_100_ polymer NPs, images are negatively stained with 1% uranyl acetate. Scale bar: 100 nm.

To investigate these hypotheses and explicitly examine the effects of using a brush topology to make hydrogels, a series of brush polymers was synthesized. These brush polymers consisted primarily of poly(ethylene glycol) (PEG, *M*_n_ 3.5 kDa, Dispersity (*Ð*) = 1.05) chains, since PEG is both hydrophilic and easily modified to allow for brush polymer formation. Polymers of varying backbone length (DP = 4, 10, 25, 50, and 100) were synthesized via ring‐opening metathesis polymerization (ROMP) of norbornene‐terminated PEG macromonomers, as ROMP allows for the synthesis of well‐defined polymer architectures to aid in the investigations of structure‐property relationships.^[^ ^21^, ^22^, ^23^ ^]^ Polymers with DP = 25, 50, and 100 were expected to form brush‐like structures in solution based on previous morphology studies of branched polymers, while the DP = 4 and 10 polymers served as controls expected to exhibit chain behavior more similar to conventional star‐polymers.^[^ ^24^ ^]^ Polymerizing star polymers and brush polymers via the same macromonomer provides us a means to explore the polymer topology's influence on the resulting hydrogels, without the concern of nonparallel comparisons. Postpolymerization, the PEG brush terminal hydroxyl groups were tosylated; batches of each polymer were then modified to express either aldehyde or hydrazide functionalities by substitution with appropriate reagents (**Figure** **1**). Using the same polymer as a precursor to both modified versions ensured accurate crosslinking group stoichiometry when mixing the two modified polymers to form a gel. Furthermore, the use of a bimolecular “A+B” system avoided the creation of ineffective intramolecular self‐loops that may form in a unimolecular system.^[^ ^25^, ^26^ ^]^ The well‐studied hydrazone formation reaction between aldehyde and hydrazide groups provided a simple system to crosslink the brush polymers into gels, as it takes place under mild conditions, and does not require any additional catalysts or external stimuli (such as high temperatures or UV light) to initiate gelation.^[^ ^27^ ^]^

Typical values of *Ð* for crude star/brush polymers ranged from 1.1 to 1.4 as measured by gel permeation chromatography (GPC, molecular weights of polymers are summarized in Table S1 in the Supporting Information), and functionalization of each polymer showed near‐quantitative yield by NMR (Figures S2–S5, Supporting Information). As a consequence of the narrow weight distribution, near‐complete monomer conversion (Figure S6, Supporting Information), and high yield of chain‐end functionalization, the branch functionality of each polymer was considered to be equal to the polymer's degree of polymerization. Brush polymers were found to form discrete globular nanostructures in aqueous solution. As determined by dynamic light scattering (DLS), the Z‐average hydrodynamic diameter ranged from 15 to 38 nm for polymers with degrees of polymerization from 25 to 100. The brush polymer nanoparticles were also imaged by transmission electron microscopy (TEM), with dry‐state diameters of 17.2 ± 1.3 and 23.3 ± 2.7 nm for aldehyde modified DP_50_ and DP_100_ brush particles, respectively (additional TEM images, Figure S7, Supporting Information). All Z‐average hydrodynamic diameters and dry‐state diameters of polymers are summarized in Table S2 in the Supporting Information. These values and morphologies are in good agreement with prior literature on similar polymer architectures consisting of a hydrophobic polymer backbone modified with a dense array of hydrophilic brushes.^[^ ^28^, ^29^ ^]^

**Figure 2:**
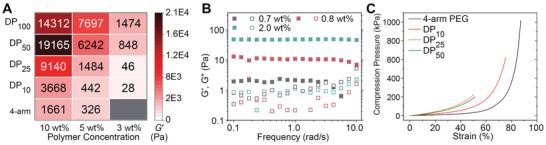
A) Storage moduli of hydrogels crosslinked by polymers with different DPs at 3, 5, and 10 wt%. B) Storage modulus (*G*′, filled squares) and loss modulus (*G*″, empty squares) as a function of angular frequency (*ω*) of DP_100_ polymer gels at low polymer concentrations. C) Stress–strain curves of PEG hydrogels crosslinked by polymers with different DPs at 10 wt%.

To investigate the influence of different polymer design parameters on the resulting hydrogels’ mechanical behaviors, the elastic moduli of the polymer networks were measured at three different polymer concentrations (3, 5, 10 wt%) for each backbone DP. Aldehyde and hydrazide modified polymers were first dissolved separately in Milli‐Q water. The solutions of complementary species were then combined, and the mixed polymer solution was injected into a silicone mold and left overnight before further characterization. A frequency sweep test (0.1–100 rad s^−1^, 2% strain, 25 °C, Figure S9, Supporting Information) was conducted for each gel. Successful gelation was characterized as having a storage modulus (*G*′) larger than the loss modulus (*G*′′) at 10 rad s^−1^ (i.e., Tan *δ* > 1). Samples that failed to form gels are marked grey in the storage modulus heatmap (**Figure** **2**A) where the value of *G*′ for each hydrogel is also labeled. These data show that while brush polymers formed gels across all polymer concentrations tested, the 4‐arm PEG could only form a gel for polymer concentrations above 5 wt%. In addition, the intermediate DP_10_ star polymer and low DP brush polymer DP_25_ could form gels at 3 wt%, but only with poor mechanical properties as evidenced by their low values of *G*′. The ability of large *f* (*f* > 25) brush polymers to form gels at lower concentrations than the star polymers is attributed to a lower overlap concentration (C*) for brush polymers than for the 4‐arm PEGs (C* ≈ 3*M*_w_/4*πN*_A_ *R*_g_^3^, where *M*_W_ and *N*_A_ are the molar weight of polymer and the Avogadro's constant, respectively).^[^ ^30^ ^]^ Because of the lower overlap concentration, at a given polymer concentration, the reactive groups of the brush polymers are more likely to be near a bonding partner, promoting gelation. We also hypothesized that the larger dimensions of the brush polymers would also lead to a significant decrease in the critical percolation concentration, *C*_0_, compared with the 4‐arm star polymer. To test this hypothesis, rheological measurements were conducted with DP_100_ brushes, which had the largest *R*_g_ in our study (dry‐state diameter of 24 nm by TEM; solvated diameter of 38 nm by DLS), at different wt% to determine *C*_0_. Remarkably, DP_100_ was able to form gels as low as 0.7 wt% (Video S1 (Supporting Information), 0.7 wt% DP_100_ hydrogel). This result is similar to oligo‐tetra‐peg hydrogels reported previously (*C*_0_ = 0.6 wt%), which involved a two‐step gelation process where maleimide and thiol group modified oligo‐tetra‐PEG clusters (*R*_h_ ≈ 90 nm) were preassembled before being crosslinked into a gel network in a second step.^[^ ^8^ ^]^ In contrast, the unimolecular brush polymer nanoparticles examined here offer a single‐step process to achieve low polymer content hydrogels, which makes this gel formulation amenable to different processing methods to achieve different macroscopic hydrogel architectures (see below).

The storage modulus heatmap also shows that gels made with brush polymers were much stiffer than 4‐arm PEG gels, as evidenced by significantly higher *G*′, even though the chemical composition of each combination was identical at a given polymer wt%. One contribution to this increased shear modulus is the higher functionality of the brush polymers. The modulus of the network is expected to increase with increasing functionality of the network components as predicted by phantom network theory (*G* ∝(*f* − 2)/*f*) for tree‐like networks.^[^ ^31^ ^]^ However, when the polymer enters the brush architecture regime, i.e., when DP is larger than 25, the *G*′ of brush polymer gels was significantly higher (5‐ to 15‐fold) than the 4‐arm PEG, which cannot be attributed to an increase in functionality alone. This unusual increase in the stiffness was therefore hypothesized to arise as a result of the unique architecture of the brush polymers. The crowding of side chains along the backbone leads to an extension of the conformations of both the backbone and the side chains, yielding a more rigid structure than typical flexible linear or star polymers in more coiled conformations.^[^ ^32^ ^]^ Therefore, the synergistic effect of high branch functionality and the rigid nature of brush polymers allows a mechanically stiff gel network to be formed.

In addition, the relationship between the *G*′ of a gel and the DP of the constituent brush polymers was found to depend on the weight percentage of polymer used. At 3 wt% and 5 wt%, *G*′ increased monotonically with increasing DP. However, at 10 wt%, the *G*′ of the gel composed of DP_100_ brushes was actually slightly lower than that for a gel made with DP_50_ brushes. Additional rheological tests of smaller DP_50_ at high wt% (15 and 20 wt%) showed similar trends, although not as pronounced as DP_100_ (Figure S10, Supporting Information). It is hypothesized that this anomaly at high wt% and DP is due to the generation of heterogeneous defects during the rapid gelation when the polymer concentration is far higher than C* (see below). Although brush polymers gels exhibited an increased shear modulus, compression tests (Figure 2C) indicated that high DP brush gels were also weaker and failed at a lower strain compared to the 4‐arm PEG or star polymer DP_10_ gels. Both DP_25_ and DP_50_ hydrogels broke at a compressive strain of ≈ 50% at a stress of ≈ 200 kPa, whereas the 4‐arm PEG hydrogel remained intact at over 80% strain and 1000 kPa. This is again attributed to the architecture of the brush polymers and the fact that the polymer chains in the gel network are already extended prior to gelation. Nevertheless, the tunability of the hydrogel stiffness (100 Pa to 20 kPa) and reasonable strength (200 kPa) are in the range of many human soft tissues and organs,^[^ ^33^, ^34^ ^]^ indicating that brush polymer gels are still suitable for many soft material applications.

**Figure 3:**
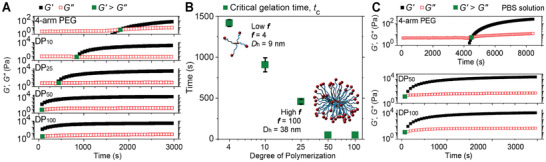
A) Gelation kinetics of hydrogels crosslinked by 4‐arm PEG, DP_10_, DP_25_, DP_50_, and DP_100_ at 10 wt% and B) their critical gelation time, *t*_c_. C) Gelation kinetics of 10 wt% 4‐arm PEG, DP_50_, and DP_100_ in PBS.

To have a better understanding of the relationship between the gelation process and polymer design, the gelation kinetics of polymer hydrogels were studied using five different macromonomer DPs. Gelation kinetics for each polymer (at 10 wt%) were evaluated by monitoring the shear modulus throughout gelation (**Figure** **3**A). Critical gelation time, *t*_c_, was defined as the point at which *G′* became larger than *G″*. It was found that the *t*_c_ of each hydrogel combination was inversely correlated with the DP of the selected polymer (Figure 3B). Typically, higher DP brush polymers formed elastic networks almost instantly (i.e., *t*_c_ was reached before the rheological test began), while the 4‐arm polymer required over 20 min to gel and was still very soft after 1 h (*G′* < 100 Pa) in water; gelation was even slower in PBS (*t*_c_ > 1 h) as a result of the increased pH slowing the hydrazone formation rate (Figure 3C).^[^ ^27^ ^]^ Furthermore, the gelation of DP_50_ and DP_100_, in which *t*_c_ times were still within 1 min, were not as delayed in the PBS solution as the 4‐arm PEG, demonstrating the potential for brush polymers to be used as fast gelation materials in biologically relevant contexts. Interestingly, the DP_100_ hydrogel formulated in PBS exhibited a stiffer structure than in water (Figure 3A,C), consistent with the hypothesis that the structural defects generated during the ultrafast gelation weaken the network's stiffness. To further investigate the brush polymer gelation process, the gelation kinetics were also evaluated for high DP brush polymers (DP_50_ and DP_100_) in more dilute solutions (5 wt%, Figure S11, Supporting Information). A similar trend was observed with the DP_100_ gel solidifying approximately two times faster than the DP_50_ gel; notably, both brush polymers still formed gels in less than 10 min even at this lower concentration (240 ± 15.3 vs 520 ± 20.4 s).

The fast gelation rate of BBPs is attributed to two factors. First, the increased *f* results in a lower critical extent of the crosslinking reaction (*ρ*_c_) according to Flory–Stockmeyer Theory (*ρ*_c_ = 2/*f)*.^[^ ^35^ ^]^ Thus, a DP_100_ brush polymer can potentially form a fully percolated gel at *ρ* = 0.02, while 4‐arm PEG can only form a percolated gel network when *ρ* reaches 0.50. In conventional hydrogels, gelation kinetics are highly dependent on the concentration of crosslinking groups. To adjust the gelation kinetics, changes must be made to the polymer concentration or crosslinking density, which usually leads to unexpected results such as increased structural defects. In contrast, the use of nanoparticle‐sized brush polymers results in fast gelation and allows for tuning the gelation rate through architectural design without adjusting the chemical composition of the gel. In other words, the brush polymer DP, can be used as a handle in controlling the gelation process. It is also important to note that the ultrafast gelation may lead to steric blocking that prevents the crosslinking of all functional groups as well as more heterogenous structures as a result of sudden gelation at the interfaces between the two polymer solutions. This steric blocking could explain why the high DP brush polymer gel (DP_100_) yielded a softer structure than the low DP brush polymer (DP_50_) at high polymer concentration (10 wt%).

**Figure 4:**
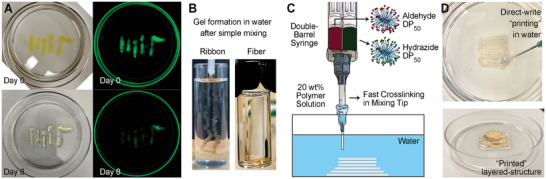
A) Direct gelation in water by injecting the mixture of 10 wt% DP_50_ polymer FITC containing solution at the bottom of a petri dish. Images on the right were captured by a fluorescent imager (exposure time 1.7 s, fluorescein filters were applied). B) Hydrogel ribbon and fiber formed by injecting 10 wt% DP_50_ polymer solution after simple mixing. C,D) Schematic concept and images of direct gelation in water by extruding 20 wt% DP_50_ polymer through a double‐barrel syringe, where mixing and gelation occurred during extrusion within the mixing tip. (Video of extrusion in the Supporting Information.)

The near instantaneous gelation of high DP brush polymers suggests their potential use as injectable hydrogels for medical applications such as therapeutic implants. To determine the potential for future investigation of brush polymers in injectable materials, direct gelation was performed under water. Precursor solutions containing 10 wt% DP50 polymer solutions and trace amounts of a FITC dye (for better visualization) were mixed, siphoned into a pipette, and then immediately injected into a water filled petri dish. Upon injecting, coherent hydrogel structures were formed at the bottom of the dish (**Figure** **4**A; Video S2, Supporting Information). The injected hydrogel structures were stable, and no noticeable physical change (except the diffusion of the FITC) was noted after 8 days immersed in water. Ribbon‐ and fiber‐like gel structures were also fabricated in the same manner by extruding the gels through a capillary piston or a needle, respectively (Figure 4B). Direct formation of gels via injection of 20 wt% precursor solutions into water could even be achieved using a double‐barrel syringe where samples were mixed in the needle and gelation occurred rapidly in the mixing tip (Figure 4C). As a result, gel structures could be deposited in a layer‐by‐layer fashion, retaining their extruded layered structures after the fabrication was complete (Figure 4D; Video S3, Supporting Information). More sophisticated structures could in principle be generated in future works with more complex additive manufacturing equipment, but this simple proof‐of‐concept shows that the gelation of these brush particles is rapid enough to enable the construction of hydrogel architectures directly in aqueous solutions.

**Figure 5:**
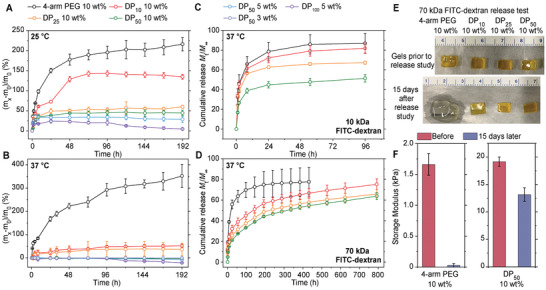
Swelling kinetics monitoring of hydrogels crosslinked by polymers with different DPs and wt% at A) 25 °C and B) 37 °C. Encapsulated FITC labeled dextran (10 kDa, (C) and 70 kDa, (D)) release profile of hydrogels crosslinked by polymers with different DPs and wt%. E) Images of hydrogels containing 70 kDa FITC labeled dextran before and 15 d after the dye release study. F) Corresponding rheological measurements of 10 wt% 4‐arm and DP_50_ PEG hydrogels after 15 d immersed in PBS.

An additional requirement for many soft gel applications, particularly in the medical field, is dimensional and mechanical stability in an aqueous environment (e.g., for long‐term drug delivery).^[^ ^7^ ^]^ For instance, a major concern regarding implanted hydrogels is the potential damage caused by swelling of the implants. In this regard, we further investigated how the brush topology can influence the swelling behavior of resulting hydrogels and compared the differences between brush polymer gels and star polymer gels during the swelling process. Swelling studies of crosslinked 4‐arm PEG, DP_10_, DP_25_, and DP_50_ hydrogels were performed at a concentration of 10 wt%. Lower polymer concentration hydrogels (3 and 5 wt% DP_50_ and 5 wt% DP_100_) were also included to examine the concentration dependent swelling behavior of brush polymer gels. For this study, all hydrogels were fabricated in phosphate buffered saline (PBS) to mimic the bodily environment, and the degree of swelling was monitored by measuring the mass of the swollen gels at predetermined time points. Tests were performed at both room temperature (25 °C) and body temperature (37 °C) for 8 d. Surprisingly, although the chemical composition of each polymer gel at an equal polymer concentration was identical, the swelling behaviors of these gels were dramatically different. Under both conditions, the 4‐arm PEG hydrogels swelled more aggressively than other brush polymer gels; the volume of 4‐arm PEG gels doubled (as determined by the wet mass change of hydrogels) within the first 8 h at both temperatures being examined and increased by ≈ 220% and 350% after 8 d at 25 and 37 °C, respectively (**Figure** **5**A,B). In contrast, gels made from DP_50_ (10 wt%) swelled only 44.6% and 1.75% over the same time period at 25 and 37 °C, respectively, and this restrained swelling was also observed in gels constructed from brush polymers with different DPs. The restrained swelling behavior of brush polymer hydrogels is attributed to the fact that the polymer chains are already forced into an extended conformation prior to gelation as a result of steric constraints introduced by the brush architecture.

Unsurprisingly, the polymer wt% in a gel also influences the swelling of brush polymer hydrogels, as lower polymer concentration gels would swell less than the higher ones (5% and 10% DP_50_ gels swelled 28.8% and 44.6% at 25 °C, respectively). Interestingly, hydrogels composed of DP_50_ brushes showed near nonswelling behavior—wet mass changes were all less than 4% throughout the 8‐d swelling period at 37 °C for all polymer wt% tested (3, 5, and 10 wt%). Because these same samples did exhibit a small amount of swelling at 25 °C, we hypothesize that the lack of observed swelling 37 °C is due to a thermoreversible syneresis effect that counteracts some of the swelling induced by osmotic pressure. This syneresis effect has previously been observed in other PEG hydrogels, where water was expelled out of a PEG network at elevated temperatures.^[^ ^36^ ^]^ Because the swelling of these gels is restrained due to the brush polymer topology, the syneresis effect at this slightly higher temperature results in a near zero change in gel volume overall. If both this hypothesis and the previous observations regarding brush networks restrain the swelling of the gels beyond a limited amount, we would further hypothesize that temperatures above 37 °C could even cause a slight shrinking of the gel. Indeed, when a swelling test was conducted at 50 °C (Figure S12A, Supporting Information), the 10 wt% DP_25_ and DP_50_ gels shrunk by 4% and 11%, respectively, after being immersed in solution for 8 d. The brush polymer gels’ degree of swelling as a function of temperature also shows the syneresis effect is somewhat mitigated with an increase in the polymer's DP (Figure S12B, Supporting Information), further indicating the potential for brush architectures as a unique tool to tune gel behavior without needing to change their composition.

Brush polymer hydrogels also showed exceptional stability in the swelling studies as evidenced by the slow erosion rate of most of the gels examined. While the DP_100_ gels began to degrade (small pieces of hydrogel were observed in the solution) after 4 d of swelling and exhibited ≈ 20% mass loss after 8 d in 37 °C PBS solution (Figure 5B), no obvious degradation was seen in other brush polymer gels (the integrity of each hydrogel piece can be confirmed through the images of swollen gels in Figure 5E), indicating the added stability of brush polymer architectures in hydrated environments.

Another potential application of injectable hydrogel scaffolds is for use in sustained drug delivery, where control over diffusion kinetics is a desirable property, and an ideal hydrogel drug delivery platform should release its contents at a steady rate. It is therefore critical to also understand how this brush polymer affects the fundamental diffusion behavior of both small and macromolecules within the gel. Given the nonswelling nature of brush polymer hydrogels and the greater structural congestion of the brush polymer building units, it was hypothesized that these gels would have a smaller mesh size and exhibit steadier molecular diffusion than 4‐arm PEG gels.^[^ ^37^ ^]^ In order to test this hypothesis, fluorescent dye modified dextran polymers, FITC_10kDa_ (Dextran molecular weight 10 kDa, *R*_h_ ≈ 2.6 nm) and FITC_70kDa_ (Dextran molecular weight 70 kDa, *R*_h_ ≈ 6.5 nm), were used to investigate the release profiles of encapsulated molecules of different sizes. The 4‐arm PEG hydrogel released over 70% of FTIC_10kDa_ and nearly 50% of FTIC_70kDa_ within the first 24 h (Figure 5C,D). However, for the DP25‐100 brush polymers, the initial release was increasingly mitigated with increased DP, and the DP_50_ (10 wt%) only released 38.8% and 15.0% of FITC_10kDa_ and FITC_70kDa_, respectively, in the initial 24 h period. Furthermore, the release half‐lives in DP_50_ gels were 55 and 336 h for 10 and 70 kDa dextran, respectively, which constitutes half‐lives that are 28‐ and 15‐fold longer than the 4‐arm PEG gels. It is also important to note that the 4‐arm PEG hydrogel lost all integrity after ≈ 20 d in the PBS buffer (complete degradation of gel structure), while the brush polymer gels were able to continue releasing FITC_70kDa_ at a steady rate (≈ 1% per day, DP_50_ at 10 wt%). Additionally, due to the nonswelling behavior of brush polymer gels at 37 °C (Figure 5E), brush polymer hydrogels could also maintain their mechanical properties after an extended period of time in an aqueous environment. Rheological measurements showed that the DP_50_ gel still possessed 70% of its original shear modulus, while the modulus of the 4‐arm PEG hydrogel was only 4% of its original value (Figure 5F; Figure S13, Supporting Information). The swelling and diffusion study demonstrated the advantages of brush polymer gels as potential long‐term molecule delivery devices. Although the focus of this work is an understanding of the basic structure‐property relationships of these gels, a preliminary biocompatibility evaluation of brush polymer hydrogels was conducted. Cell viability was measured after incubating hydrogels with three kinds of cells, including human embryonic kidney 293 cells (HEK 293), ovarian cancer cells (SKOV3), and nonsmall cell lung cancer cells (NCI‐H358). No noticeable toxicity was observed among 4‐arm PEG gels and brush polymer gels with identical chemical composition, when compared with the negative control groups after cocultured for 3 d (Figure S14, Supporting Information).

## 3 Conclusion

Gelation is a complex process that can be influenced by multiple factors, including the branch functionality of each building unit, extent of chain entanglements, and overall size of the selected polymers. Conventional strategies involving large linear biopolymers or multifunctional crosslinkers to tune the gelation process are a valuable tool in gel design, but can unfortunately introduce inherent challenges due to their severe chain entanglements (e.g., difficulty in fully dissolving the polymers, high viscosity of the resulting solutions).^[^ ^18^ ^]^ In contrast, with their absence of chain entanglements, brush polymers offer a simple yet effective tool to tune the gelation process as well as the properties of the resulting gels. In this study, we investigated how the unique architecture of brush polymers can influence the gelation process, including the gelation kinetics, stiffness, and following swelling process. We further demonstrated a fast‐forming, nonswelling network by employing brush polymers as building blocks. This approach, in principle, allows for the tuning of the hydrogel's properties, including stiffness and gelation kinetics, over a wide range by simply altering the DP without any change in the chemical composition, further expanding the toolbox of design handles for preparing hydrogels with desired properties. These capabilities could also enable future studies to better understand the gelation process by separating the influence of complex chain entanglements from the other polymer design parameters. In addition to the evasion of chain entanglements, the strong steric hinderance between adjacent side chains on brush polymers also allows for nonswelling behavior under physiological conditions and, therefore, a prolonged and steady diffusion of encapsulated macromolecules, indicating these gels’ potential as injectable delivery devices in soft tissue. Collectively, this work offers a new building block for hydrogel structural design and provides a strategy for the synthesis of advanced polymer materials, which can benefit both future theoretical gelation studies and injectable medical devices.

## Conflict of Interest

The authors declare no conflict of interest.

## Supporting information

Supporting Information

Supplemental Video 1

Supplemental Video 2

Supplemental Video 3

## Data Availability

Research data are not shared.
