# Supplementary material for: Design and Synthesis of Quick Setting Nonswelling Hydrogels via Brush Polymers
Source: Adv Sci (Weinh). 2021 Jun 20;8(16):2100968. doi: 10.1002/advs.202100968 (PMC8373163; doi:10.1002/advs.202100968)
Supplement: Supplementary file 1 — Supporting Information [file ADVS-8-2100968-s002.pdf]

WILEY-VCH

© 2021 Wiley-VCH GmbH

ADVANCED SCIENCE
Open Access

Supporting Information

for *Adv. Sci.*, DOI: 10.1002/advs.202100968

Design and Synthesis of Quick Setting Non-Swelling Hydrogels via Brush Polymers

*Fei Jia, Joshua M. Kubiak, Michika Onoda, Yuping Wang, and Robert J. Macfarlane**

((Supporting Information can be included here using this template))

Supporting Information

Design and Synthesis of Quick Setting Non-Swelling Hydrogels via Brush Polymers

*Fei Jia, Joshua M. Kubiak, Michika Onoda, Yuping Wang, and Robert J. Macfarlane**

This PDF file includes:

Supplementary Materials and Methods

Scheme S1. Synthesis of PEG monomer.

Scheme S2. Preparation of Grubbs 3rd catalyst and ROMP of N-PEG-OH.

Scheme S3. Tosylation of hydroxy groups terminated PEG brushes.

Scheme S4. Synthesis of aldehyde-terminated brush PEGs.

Scheme S5. Synthesis of acylhydrazine-terminated brush PEGs.

Figure. S1. MALDI-MS of hydroxy terminated PEG (3.5 kDa) and N-PEG (~3.7 kDa) macromonomer using sinapinic acid matrix.

Figure. S2-S5. Representative NMR spectra of modified brush PEGs.

Fig. S6. Additional TEM images of brush PEGs.

Fig. S7-S11. Rheological measurements of polymer hydrogels.

Fig. S12. Swelling test of hydrogels at 50 ºC and hydrogel over a temperature range from 25 °C to 50 °C.

Fig. S13. Rheological measurements of swollen polymer hydrogels.

Fig. S14. MTT cell viability for 4-arm PEG gel and DP_{50} gel.

Table S1. Molecule weight and dispersity of polymers measured by GPC.

Table S2. R_h and dry-state diameter measured by DLS and TEM, respectively.

References. (1-5)

1. Materials and methods.

Heterobifunctional poly(ethylene glycol) (PEG, hydroxyl PEG amine, OH-PEG-NH_2) (M_n = 3.5 kDa, PDI < 1.04) was purchased from JenKem Technology, USA. All other materials were purchased from Sigma-Aldrich Co., VWR International LLC., or Fisher Scientific Inc. and were used without further purification unless otherwise indicated.

D_h data were acquired on a Wyatt Dyna Pro Plate Reader (laser wavelength 850 nm, light scattering angle at 90°). *N*, *N* Dimethylformamide (DMF) gel permeation chromatography (GPC) was carried out on a TOSOH EcoSEC HLC-8320 GPC system (Tokyo, Japan) equipped with a TSKGel GMH_{HR}-H, 7.8×300 mm column and RI/UV-Vis detectors, using ReadyCal-Kit PEO/PEG, M_w 232 - 941 kDa as standards (PSS-Polymer Standards Service - USA Inc). Samples with a concentration of 1.0 mg/mL were run at a flow rate of 0.5 mL/min at 50 °C using 0.05 M LiBr DMF as the mobile phase. ^{1}H and ^{13}C NMR spectra were recorded on a Bruker Avance Neo 500 MHz spectrometer (Bruker Daltonics Inc., MA, USA). Chemical shifts (δ) are reported in ppm. MALDI-ToF MS measurements were carried out on a Bruker Microflex LT mass spectrometer (Bruker Daltonics Inc., MA, USA) using a sinapinic acid matrix. The dynamic mechanical properties of the hydrogels were tested on an Anton Paar rheometer with parallel plate geometry (PP-10 probe, 10 mm diameter, flat). The compression tests of hydrogels were carried out on a dynamic mechanical analyzer (TA instruments DMA 850) at ambient temperature at a strain rate of 10%/min. For TEM analysis, samples were deposited on carbon-coated copper grids (Ted Pella Inc., CA, USA) for 5 min before excess liquid was carefully wicked away by filter paper. The grids were then stained by pipetting 10 µL of 1% uranyl acetate directly onto the grid. The stain was allowed to stay for 1 min before being wicked away. All TEM samples were imaged on a FEI Tecnai Multipurpose Digital TEM electron microscope utilizing an accelerating voltage of 120 kV.

2.1 Syntheses of norbornenyl N-hydroxysuccinimidyl ester (N-NHS) and norbornenyl PEG macromonomer (N-PEG-OH).

Scheme 1. Synthesis of PEG monomer.

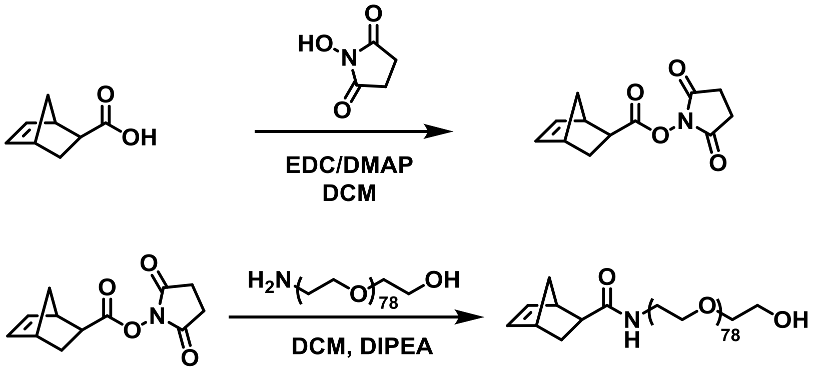

N-NHS

N-PEG-OH

Norbornenyl hydroxysuccinimidyl ester (N-NHS) was synthesized according to a previously reported method[1] (**Scheme S1**). In brief, N-NHS was synthesized by esterification reaction between norbornenyl carboxylic acid (4.0 g, 1 equiv.) and *N*-hydroxysuccinimide (NHS, 5.0 g, 1.5 equiv.) using *N*-(3-Dimethylaminopropyl)-N′-ethylcarbodiimide hydrochloride (EDC HCl, 8.9 g, 1.6 equiv.) as a coupling agent in dichloromethane (50 mL). N-NHS was purified by silica column (2:1 hexanes/ethyl acetate as eluent) to afford a white solid product with 80% yield.

^{1}H-NMR (400 MHz, $CDCl_3$): δ 6.20-6.12 (m, 2H), 3.27 (s, 1H), 3.00 (s, 1H), 2.84 (s, 4H), 2.50 (m, 1H), 2.05 (m, 1H), 1.55-1.50 (m, 1H), 1.48-1.40 (m, 2H); ^{13}C-NMR (100 MHz, $CDCl_3$): δ 171.9, 169.5, 138.8, 135.5, 47.4, 46.6, 42.0, 40.5, 31.3, 25.8.

N-PEG-OH macromonomer was obtained using a previously reported method[2]. Briefly, OH-PEG-NH_2 (2.0 g, 1.0 equiv.) and N-NHS (161 mg, 1.2 equiv.) were dissolved in anhydrous dichloromethane in a round bottom flask, to which N, N-diisopropyl ethyl amine (DIPEA, 250 µL, 2.0 equiv.) was added via a pipette. The reaction mixture was allowed to stir overnight at room temperature. Next, the reaction mixture was concentrated and precipitated in 100 mL of ice-cold diethyl ether 3x. The resulting white precipitant was dried in vacuo (2.0 g obtained, yield 93%). The modification of PEG was confirmed by using MALDI-ToF (**Figure S1**).

2.2 General method for the synthesis of bottlebrush polymers.

Scheme 2. Preparation of Grubbs 3rd catalyst and ROMP of N-PEG-OH

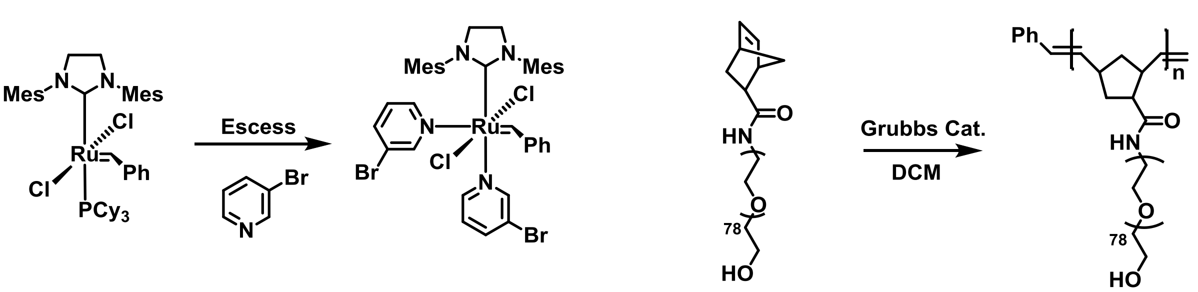

3rd generation Grubbs' catalyst was synthesized based on a published protocol[3]. In brief, 2nd generation Grubbs's catalyst (500 mg) and excess 3-bromopyridine (1.0 mL) were vortexed in 15 mL centrifuge tube. The reaction mixture was then precipitated in anhydrous pentane (10 mL) 3x. The resulting green powder was dried in vacuo and stored under -20 ºC.

Star/brush PEG were polymerized using ring-opening metathesis polymerization (ROMP). A typical polymerization process for brush PEG is as follows (example given for the DP_{100}). All apparatuses and chemical compounds were first transferred to a nitrogen glove box. Then, N-PEG-OH (200 mg) were dissolved in anhydrous DCM (0.6 mL) in a 5 mL Schlenk flask, and the catalyst (0.59 mg) was dissolved in DCM (0.2 mL) in a 1.5 mL micro centrifuge tube; this catalyst solution was then injected into the flask via a micro syringe. The reaction mixture was cooled to -20 ºC by using an ice-salt bath and stirred for 2 h. At the end of the reaction, excess ethyl vinyl ether (EVE, 0.2 mL) was added to the mixture to ensure polymerization termination, and the reaction was stirred for an additional hour. The mixture was then concentrated and precipitated into ethyl ether (50 mL, 3×), and the precipitant was dried in vacuo.

Typical monomer conversion during ROMP using 3rd generation Grubb's catalyst was observed to be nearly quantitative, as has been reported in previous literature[4,5]. The full consumption of all macromonomers was verified using GPC measurements performed before purification (via precipitation in ether) of the polymerization product; this data is shown in **Figure 1B**.

2.3 Synthesis of terminal modified PEG polymers

Scheme 3. Tosylation of hydroxy groups terminated PEG brushes.

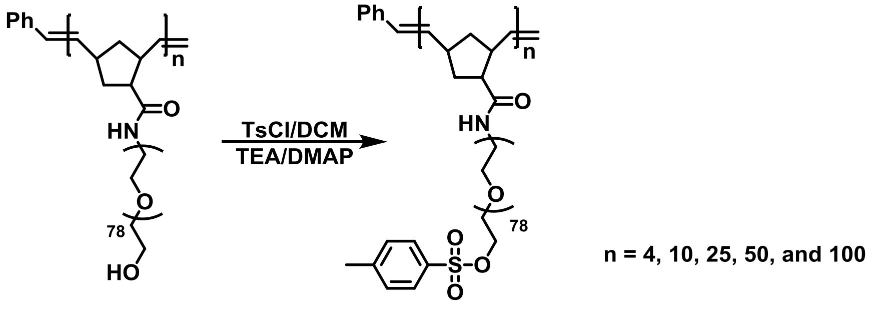

p-Toluenesulfonate-terminated PEG brushes (Ts-PEG).

Brush PEG (200 mg), 4-Dimethylaminopyridine (DMAP, 200 mg), and triethylamine (TEA, 300 µL) were dissolved in anhydrous DCM in a round bottom flask; the mixture was cooled in an ice-water bath. To this solution, 4-Toluenesulfonyl chloride (TsCl, 150 mg) dissolved in DCM was added

dropwise. The reaction mixture was stirred at room temperature overnight. Then, the polymer solution was concentrated and precipitated in ice cold diethyl ether (50 mL). The brownish precipitate was collected and dissolved in Milli-Q® water, followed by extensive dialysis against Milli-Q® water (Spectrum®, US, Molecular weight cut-off, MWCO, 6 ~ 8 kDa) for 2 days to remove small reactants. The final solution was lyophilized to yield yellowish solids (yield: 95%). The full conversion of the hydroxyl group was confirmed by the complete disappearance of the corresponding peak in ^{1}H NMR (DMSO-d_6, 400 MHz, 298 K): *δ* 7.78 (d, 2-H of phenyl), 7.48 (d, 3-H of phenyl), 4.12 (t, Ts-O-CH_2-), 2.43 (s, CH_3-phenyl-). The ^{1}H NMR spectrum of *p*-Toluenesulfonate-terminated DP_{50} is shown in **Figure S2**.

Scheme 4. Synthesis of aldehyde-terminated brush PEGs.

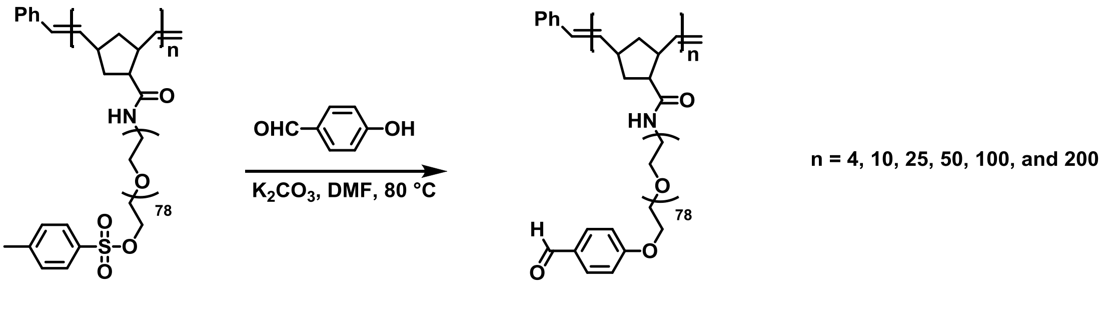

Aldehyde-terminated brush PEGs

Ts-PEG (200 mg) and 4-hydroxybenzaldehyde (200 mg) were dissolved in DMF (5 mL). To this solution, K_2CO_3 (200 mg) was added and stirred for 48 h at 80 °C. Small molecule reactants were removed by dialysis against Milli-Q® water for 48 h (MWCO = 6~8 kDa). The final solution was lyophilized to yield yellowish solids (yield: 90%) and stored at -20 °C. ^{1}H NMR (DMSO-d_6, 400 MHz, 298 K): δ 9.87 (s, -CHO), 7.86 (d, 2-H of phenyl), 7.15 (d, 3-H of phenyl), 4.22 (t, phenyl-O-CH_2-), 3.78 (t, phenyl-O-CH_2-CH_2-). The ^{1}H NMR spectrum of *p*-Toluenesulfonate-terminated DP_{50} is shown in **Figure S3**.

Scheme 5. Synthesis of acylhydrazine-terminated brush PEGs.

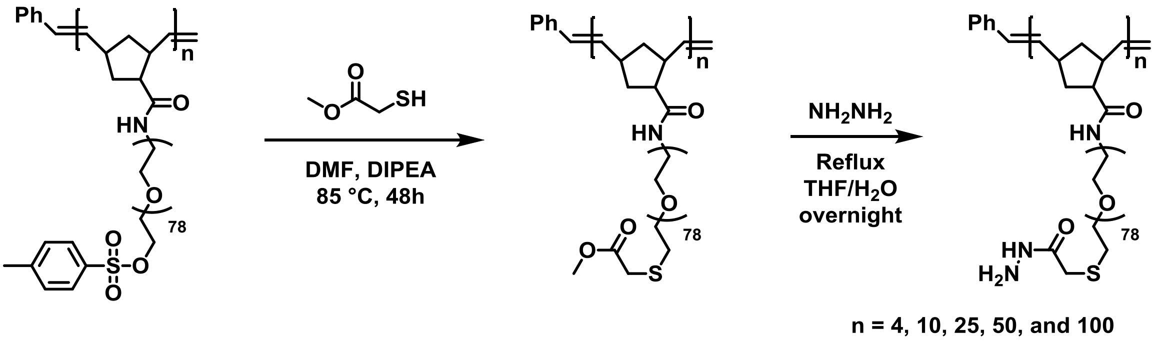

Methyl thioacetate-terminated brush PEGs (MTA-PEGs)

Ts-PEG (200 mg) and methyl 2-mercaptoacetate (200 mg) were dissolved in 1.5 mL DMF in a 2 mL centrifuge tube, followed by addition of 150 µL DIPEA. The reaction mixture was heated to 80 °C and shaken for 48 h. Then, the methyl thioacetate-terminated brush PEG was obtained by precipitating into ice-cold diethyl ether (50 mL, 3×). The resulting yellowish precipitant was then dried under vacuum. (Yield: 90%). ^{1}H NMR (DMSO-d_6, 400 MHz, 298 K): δ 9.87 (s, -CHO), 7.86 (d, 2-H of phenyl), 7.15 (d, 3-H of phenyl), 4.21 (t, phenyl-O-CH_2-), 3.77 (t, phenyl-O-CH_2-CH_2-). The ^{1}H NMR spectrum of Methyl thioacetate-terminated DP_{50} is shown in **Figure S4**.

Acylhydrazine-terminated brush PEGs

MTA-PEG (200 mg) and hydrazine monohydrate (1 mL) were dissolved in water/ tetrahydrofuran (7/3, 10 mL) in a round bottom flask. The reaction solution was heated to reflux under N_2 for 24 h and then dialyzed against Milli-Q® water for another 48 h (MWCO = 6~8 kDa) to remove excess reactants. Acylhydrazine-terminated brush PEG was then acquired as a white powder after lyophilization (Yield, 85%). ^{1}H NMR (DMSO-d_6, 400 MHz, 298 K): δ 9.61 (s, -NH-), 7.79 (d, 2-H of phenyl), 6.98 (d, 3-H of phenyl). The ^{1}H NMR spectrum of Methyl thioacetate-terminated DP_{50} is shown in **Figure S5**.

2.4 General method for R_h measurements.

R_h for each polymer was measured using a Wyatt Dyna Pro Plate Reader DLS. Measurements were carried out at a polymer weight concentration of 1 mg/mL in Milli-Q® water at room temperature. All samples were passed through a syringe filter (Nylon, 0.2 µm, VWR, US) to remove impurities prior to measurement.

2.5 General method for hydrogel formation.

Gelation was achieved by mixing the aldehyde and hydrazine modified polymer counterparts. In brief, aldehyde and hydrazine modified polymers were first dissolved in water in separate micro centrifuge tubes and sonicated for at least 2 min to ensure that the polymers were well dissolved. Then, the aldehyde modified polymer solution was mixed with the hydrazine modified polymer solution via pipette and then injected into a precut silicone mold. The reaction mixture was then sealed to prevent water evaporation and incubated under room temperature for further characterizations.

2.6 General procedure for rheology measurements.

Dynamic rheology tests for all polymer network samples were conducted 1h after the gelation process. A Peltier hood with a water cell was used for all experiments to control the temperature during measurement and to prevent sample dehydration. Dynamic oscillatory strain amplitude sweep measurements were conducted at a frequency of 10 rad/s to determine the linear viscoelastic region (LVR) (**Figure S8**); storage modulus (G′) and loss modulus (G″) were measured as a function of strain amplitude ranging from 1 to 100% on a logarithmic scale. Frequency sweep measurements were conducted at a 5% strain amplitude and angular frequency ranging from 10 to 0.1 rad/s on a logarithmic scale while monitoring G′ and G″. The determination of which samples had formed fully networked gels was determined by the relative value of G′ and G″ at 10 rad/s, 25 ℃

Gelation kinetics measurements were performed by measuring the G′ and G″ immediately after placing the polymer solution mixtures on the sample stage. Then, G′ and G″ were monitored over time by oscillatory measurements (τ = 10 Pa, ω = 10 rad/s) at 25 °C. For these tests, the polymer samples were sealed by a layer of mineral oil to prevent dehydration during the testing period.

2.7 General procedure for the hydrogel compression test.

10 mg aldehyde and hydrazine modified PEGs (DP = 4, 10, 25, and 50) were separately dissolved in 90 µL Milli-Q® water in microcentrifuge tubes (10 wt%). After the polymers were fully dissolved in water, the aldehyde modified PEG solution was the transferred into the microcentrifuge tube containing hydrazine modified PEG solution. After thorough mixing, the polymer solution mixture was injected into a silicone mold (diameter: 12 mm; height: 5 mm) and sealed. The resulting hydrogels were then cut into small cylinders with a diameter of 5mm and a height of 3 mm by using a hole punch. The compression test was carried out using a dynamic mechanical analyzer (DMA 850, TA instruments) at a strain rate of 10%/min.

2.8 General procedure for the hydrogel swelling study.

In a typical experiment, a 10 wt% or 5 wt% polymer hydrogels was prepared with phosphate buffered saline (PBS) in a micro centrifuge tube and left overnight before further testing. After the gelation, 1 mL PBS buffer was put on top of the hydrogel, and the centrifuge tube was subsequently placed in a thermomixer at 300 rpm and 25 °C, 37 °C, or 50 ºC. The PBS buffer was removed at predetermined time points, and the weights of the hydrogel and micro centrifuge tube were measured. The change of hydrogel mass was used to quantitatively evaluate the hydrogel's swelling or shrinking behavior.

2.9 General procedure for the FITC release measurements.

Fluorescent dye labeled dextran (1 wt% of 10 kDa or 70 kDa dextran) was encapsulated in the hydrogel (10 wt%, 180 µL PBS) by dissolving the dye and the polymer in PBS buffer prior to gel formation. Hydrogels were prepared by injecting the polymer mixture into silicone molds (Length: 21 mm; width: 5 mm; height: 2mm) and then sealing the molds. After gelation, each hydrogel was then cut into three pieces with same size (Length: 7 mm; width: 5 mm; height: 2mm) and gel pieces were subsequently incubated at 37 °C on a thermomixer at 300 rpm with PBS buffer placed on top of the hydrogel. By collecting and changing the PBS buffer at predetermined time intervals, the release profiles of the fluorescent dyes were calculated *via* plate reader characterization of the emission of FITC at 488 nm.

2.10 MTT assay.

The ovarian cancer cells (SKOV3) and Human embryonic kidney 293 cells (HEK 293) were grown in DMEM medium supported with 10 % heat inactivated fetal bovine serum, 1% antibiotics, and 1% L-glutamine. Non-small cell lung cancer cells (NCI-H358) were grown in RPMI-1640 medium supported with 10 % heat inactivated fetal bovine serum, 1% antibiotics, and 1% L-glutamine. All cells were incubated at 37 ºC in a humidified atmosphere containing 5 % CO_2.

The cytotoxicity of 4-arm PEG gels and DP_{50} brush polymer gels evaluated using the MTT assay. Briefly, hydrogels were pre-formed in PBS buffer at 10 wt% and were then cut into small cubes (2 mm × 2 mm × 2 mm). Cells were seeded in a 96-well plate in 100 µL medium and cultured for 24 h. The cells were then cocultured with hydrogel cubes. Cells treated with PBS were used as a negative control. After 3 days, 3-[4,5-dimethylthiazol-2-yl]-3,5-diphenyltetrazolium bromide (MTT dye, final concentration of 0.5 mg/mL) was added. The cells were incubated for 4 h and the absorbance was

measured at 570 nm using a microplate reader (Biotek Synergy HT, **Figure S14**).

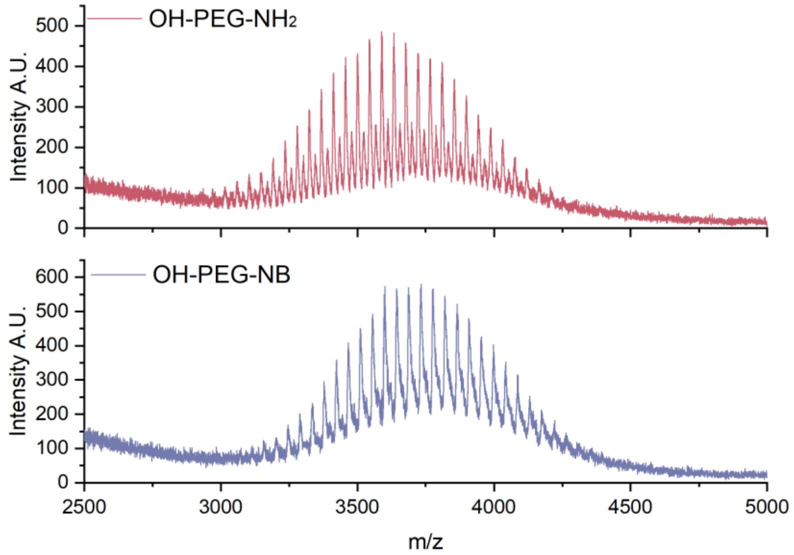

Figure S1. MALDI-MS of amine terminated PEG-OH (3.5 kDa) and NB-PEG-OH (~3.7 kDa) macromonomer using sinapinic acid matrix.

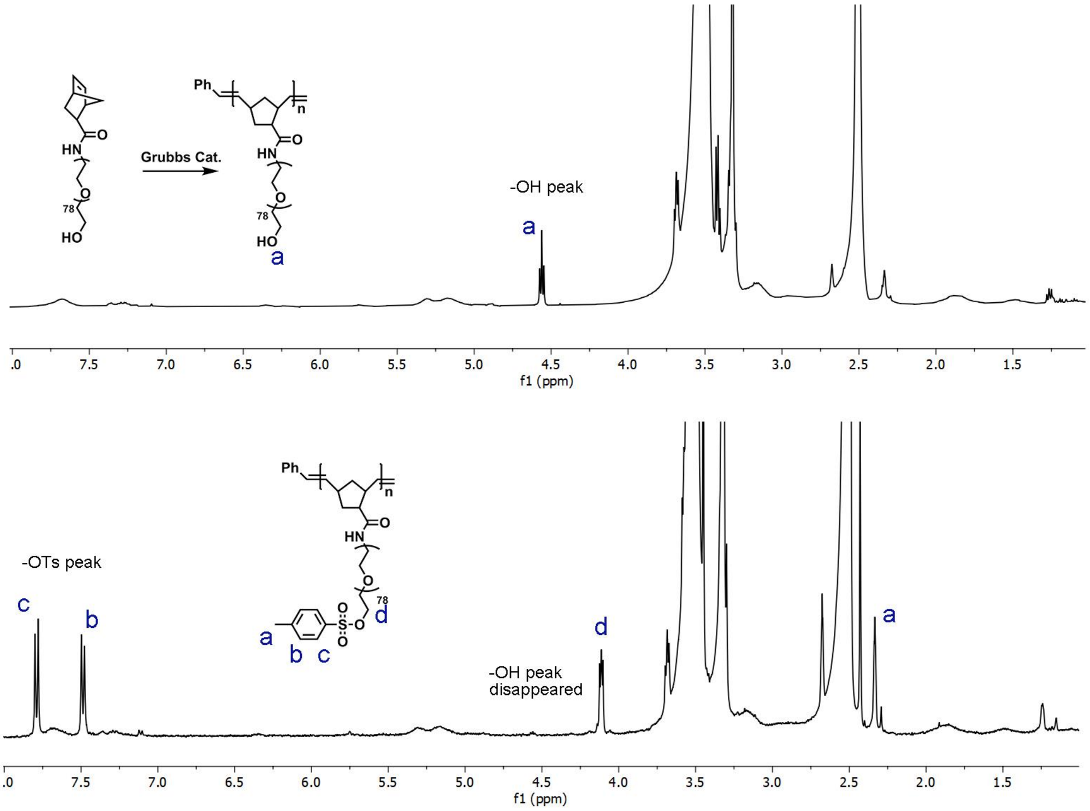

Figure S2. ^{1}H NMR spectra of (DMSO-d_6, 500 MHz, 298 K) of hydroxy terminated DP_{50} (top) and p-toluenesulfonate-terminated DP_{50}.

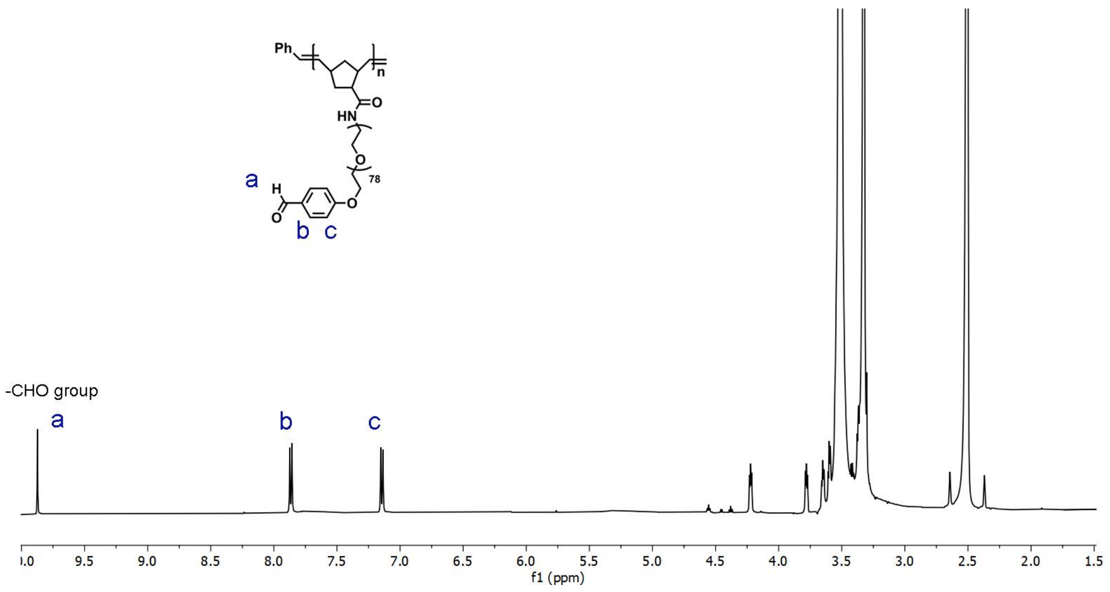

Figure S3. ^{1}H NMR spectrum of (DMSO-d_6, 500 MHz, 298 K) of aldehyde modified DP_{50}.

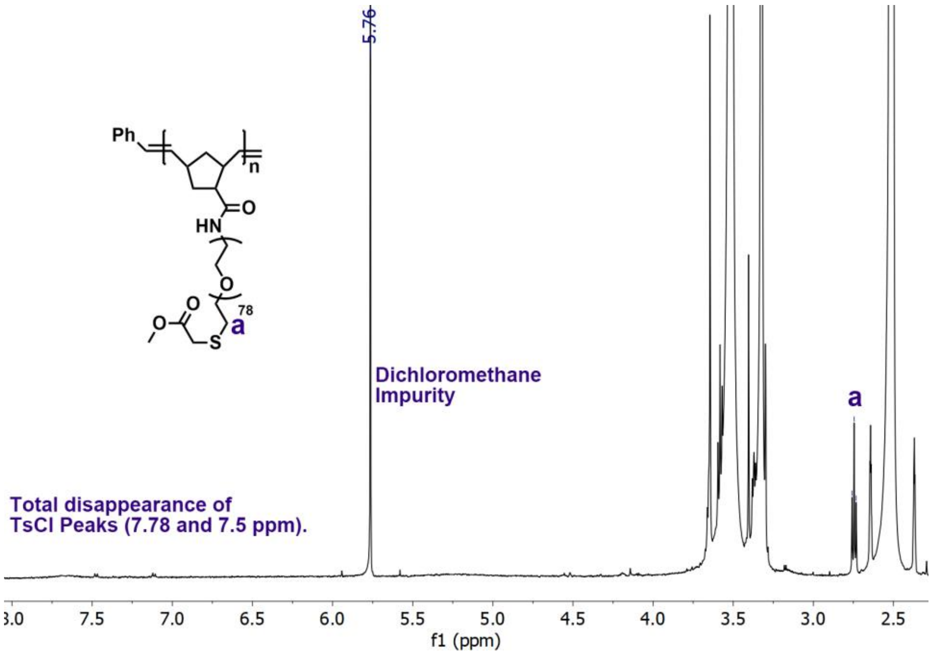

Figure S4. ^{1}H NMR spectrum of (DMSO-d_6, 500 MHz, 298 K) of Methyl thioacetate-terminated DP_{50}.

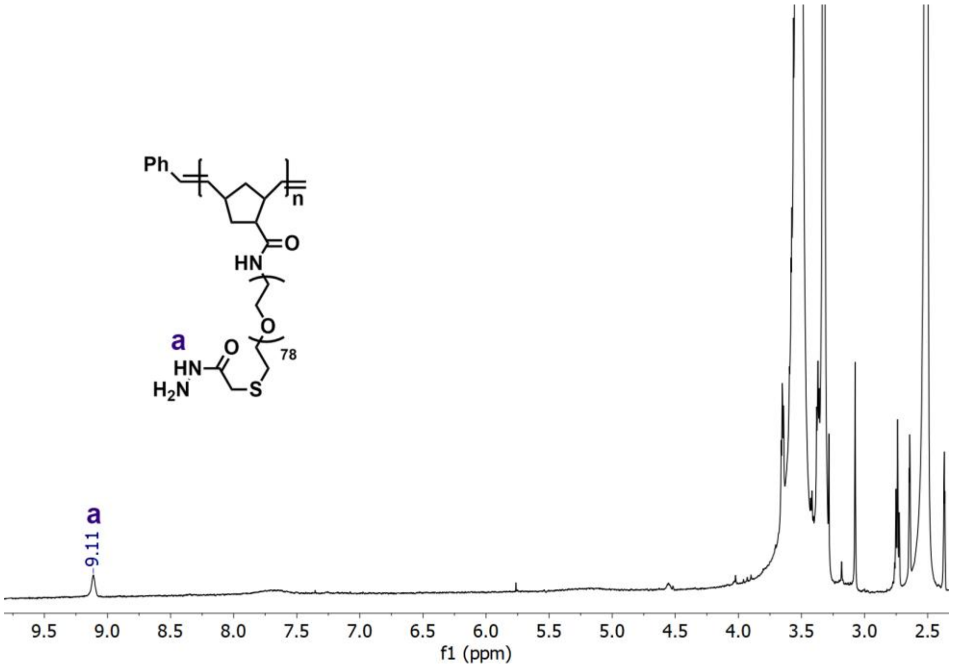

Figure S5. ^{1}H NMR spectrum of (DMSO-*amin*$_6$, 500 MHz, 298 K) of hydrazine modified DP$_{50}$.

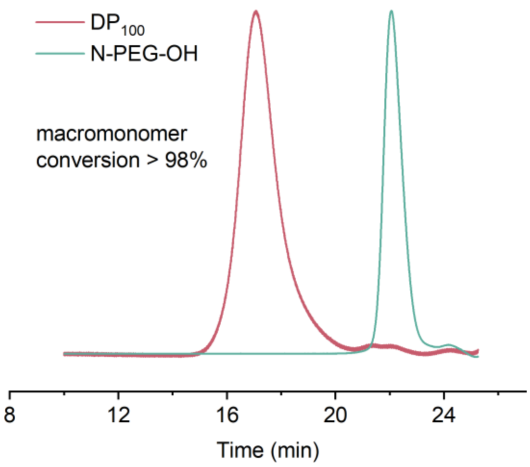

Figure S6. DMF GPC traces of brush polymer with DP 100 and macromonomer, yield calculation is based on the integration of RI signal.

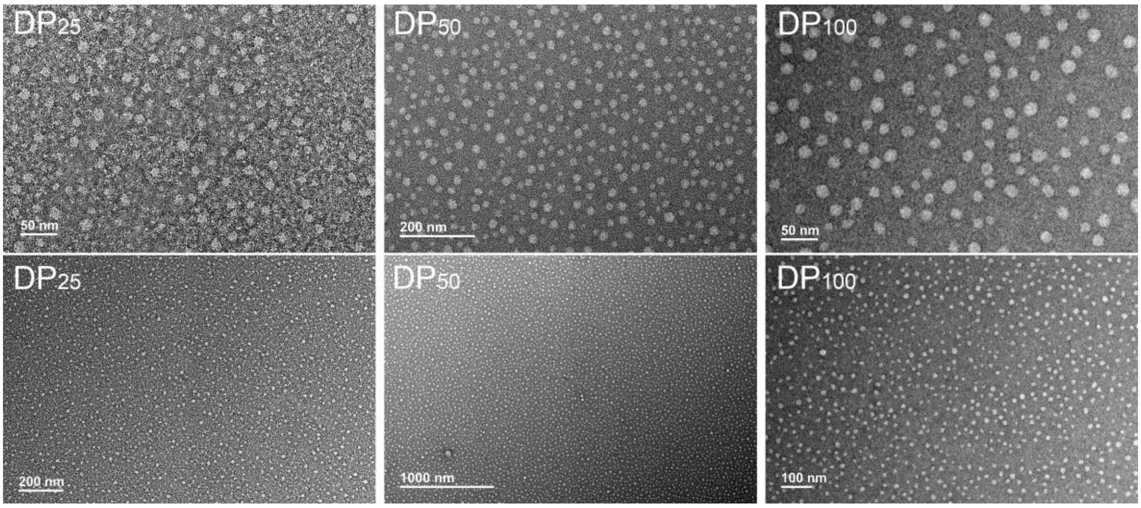

Figure S7. TEM images of brush polymers.

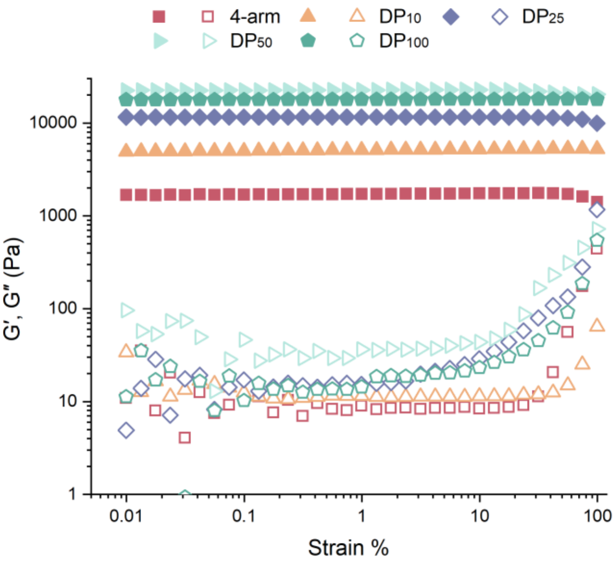

Figure S8. Strain dependent (ω=10 rad/s, 25℃, 10 wt.%) rheology measurements PEG hydrogels (4-arm, DP_{10}, DP_{25}, DP_{50}, and DP_{100},). *G'*, solid dots; *G''*, empty dots.

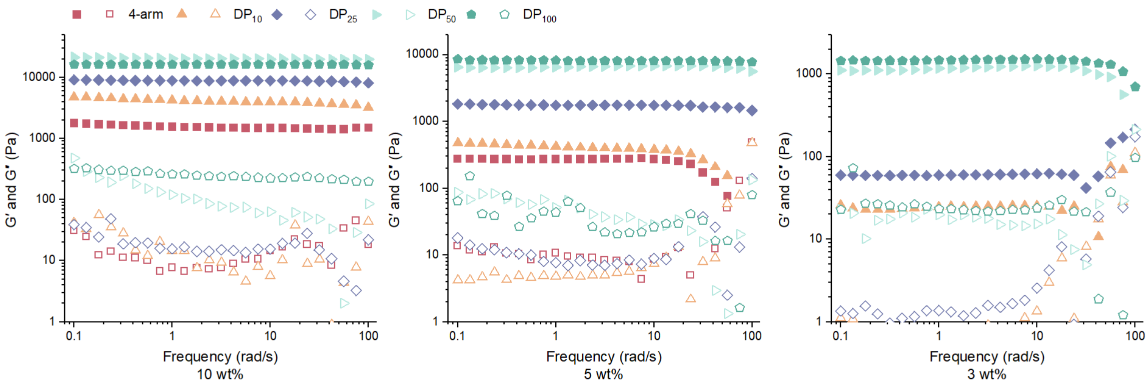

Figure S9. Frequency dependent (ϵ=5%, 25 ºC) rheology measurements of PEG hydrogels (3, 5, and 10 wt%). *G*′, solid dots; *G*″, empty dots.

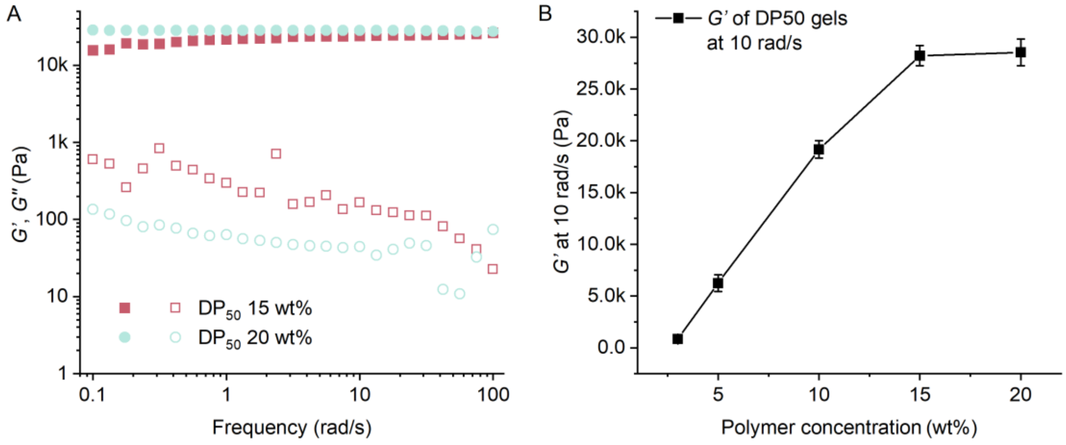

Figure S10. (A) Frequency dependent (ϵ=5%, 25 ºC) rheology measurements of DP_{50} (15, and 20 wt%). *G*′, solid dots; *G*″, empty dots. (B) *G*′ of DP_{50} as a function of polymer concentration.

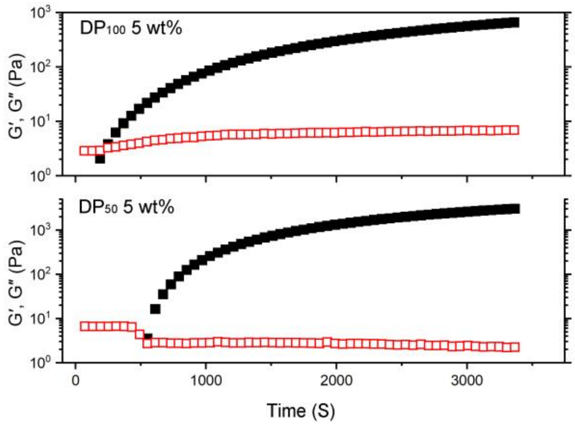

Figure S11. Gelation kinetics of hydrogels crosslinked by 5 wt% of DP_{50} and DP_{100}.

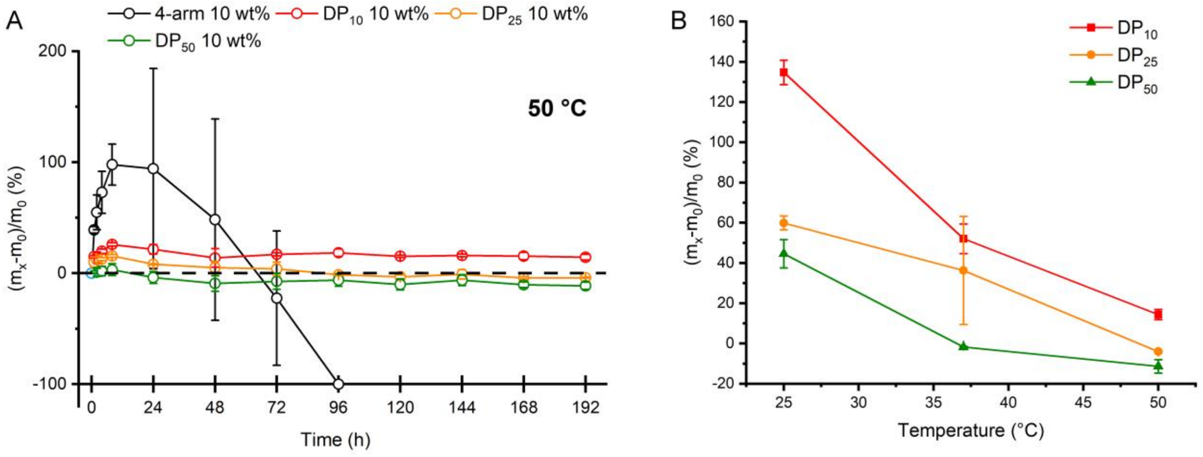

Figure S12. (A) Swelling kinetics of hydrogels crosslinked by polymers with different DPs and wt%s at 50 ºC. (B) Swelling of selected brush polymer hydrogels (10 wt%) over a temperature range from 25 °C to 50 °C.

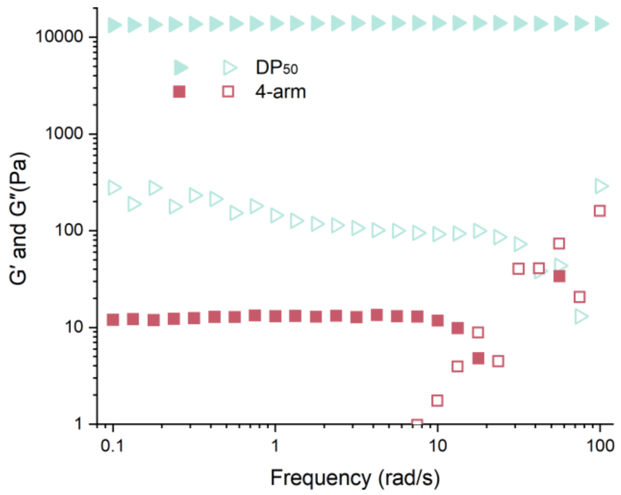

Figure S13. Frequency dependent (ϵ=5%, 25 ºC) rheology measurements of 10 wt% 4-arm and DP50 after 15-days swelling. *G′*, solid dots; *G″*, empty dots.

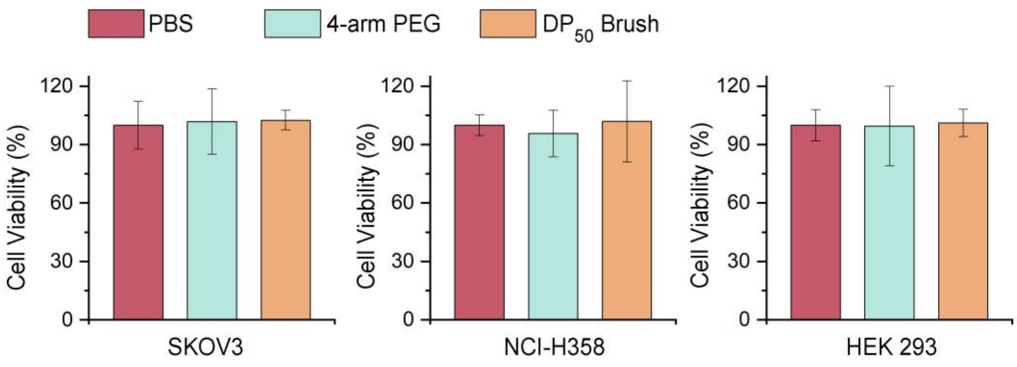

Figure S14. Cell viability of SKOV3, NCI-H358, and HEK 293 cells treated with hydrogels and controls.

Table S1. GPC Analyses for the 4-arm PEG and brush polymers Used. (M_W of brush polymers s are measured with conventional GPC and later calibrated with GPC-MALS)

Polymer	M_n (kDa)	M_w (kDa)	*Đ*
4-arm	14.6	17.5	1.2
DP_{10}	48.5	56.4	1.1
DP_{25}	121.4	148.5	1.2
DP_{50}	215.0	274.1	1.2
DP_{100}	457.7	647.6	1.4

Table S2. Hydrodynamic radius (R_h) and dry-state radius measured by DLS and TEM, respectively. Cumulant fit R_h (Z-average R_h) are reported. Dry-state radius of brush polymers, DP_{25}, DP_{50} and DP_{100}, are determined by measuring at least 50 individual nanoparticles in several TEM images

Polymer	Hydrodynamic radius, R_h (nm)	Dry-state radius (nm)
4-arm	4.6 ± 0.1	-
DP_{10}	5.8 ± 0.1	-
DP_{25}	7.9 ± 0.1	6.1 ± 0.6
DP_{50}	16.9 ± 0.4	8.6 ± 0.6
DP_{100}	18.9 ± 0.3	11.7 ± 1.3

References:

(1) Pontrello, J. K.; Allen, M. J.; Underbakke, E. S.; Kiessling, L. L. *J. Am. Chem. Soc.*

2005, *127*, 14536.

(2) Lu, X.; Watts, E.; Jia, F.; Tan, X.; Zhang, K. *J. Am. Chem. Soc.* **2014**, *136*, 10214–10217

(3) Love, J. A.; Morgan, J. P.; Trnka, T. M.; Grubbs, R. H. *Angew. Chem. Int. Ed.* **2002**,

41, 4035.

(4) Jha, S.; Dutta, S.; Bowden, N. B. *Macromolecules* **2004**, *37*, 4365–4374.

(5) Shibuya, Y.; Tatara, R.; Jiang, Y.; Shao-Horn, Y.; Johnson, J. A. *J. Polym. Sci. Pol. Chem.* **2019**, *57*, 448–455.
